# Endoscopic totally extraperitoneal approach (TEA) technique for primary ventral hernia repair

**DOI:** 10.1007/s00464-020-07575-8

**Published:** 2020-04-27

**Authors:** Binggen Li, Changfu Qin, Reinhard Bittner

**Affiliations:** 1 Department of General Surgery, Affiliated Hexian Memorial Hospital of Southern Medical University, Guangzhou, 511400 China; 2 Department of Hernia and Abdominal Wall Surgery, Beijing Chaoyang Hospital, Capital Medical University, Beijing, 100043 China; 3 I.M. Sechenov First Moscow State Medical University of the Ministry of Health of the Russian Federation (Sechenov University), Trubetskaya str., 8, b. 2, Moscow, Russia 119992; 4 Emeritus Director Marienhospital Stuttgart, Supperstr. 19, 70565 Stuttgart, Germany

**Keywords:** Primary ventral hernia, Umbilical hernia, Epigastric hernia, Endoscopic repair, Totally extraperitoneal approach

## Abstract

**Background:**

Up to now the totally extraperitoneal (TEP) technique is limited to the treatment of inguinal hernias. Applying this anatomical repair concept to the treatment of other abdominal wall hernias, we developed an endoscopic totally extraperitoneal approach (TEA) to treat primary midline ventral hernias, including umbilical and epigastric hernias, in which for mesh placement, an anatomical space is developed between the peritoneum and the posterior rectus sheath in the ventral part of the abdominal wall (preperitoneal space).

**Methods:**

Between September 2017 and December 2019 according to the selection criterions, 28 consecutive primary midline ventral hernias were repaired using TEA. After extensive endoscopic development of the midline extraperitoneal plane, which was started in the suprasymphysic area, and reduction of the hernia sac, the hernia defect was closed and a large mesh was placed in the preperitoneal position to enforce the anterior abdominal wall.

**Results:**

All operations were successfully performed without conversion to open surgery. The mean operation time was 103.3 min (range 85–145 min). Patient-reported postoperative pain was qualitatively mild with a mean pain visual analogue scale score of 1.9 on postoperative day 1. The average hospital stay was 1.9 days (range 1–3 days). Three patients developed minor complications and were treated with no long-term adverse effects. Readmissions within 30 days or hernia recurrences were not observed with a mean follow-up period of 18 months (range 10–27 months).

**Conclusion:**

In selected cases, TEA is a safe and feasible minimally invasive alternative in treating primary ventral hernias. This technique preserves the anatomical and physiological structure of the abdominal wall and may significantly reduce trauma and postoperative complications. Additionally, anti-adhesion-coated meshes and fixation tackers are not required, thus being cost-effective. Further studies are necessary to proof the true clinical efficacy in comparison to well-known alternative techniques.

The totally extraperitoneal (TEP) technique for repair of inguinal hernia was first reported in 1992 by Dulucq [1] and has since been continuously refined and standardized. In fact, it has become one of the gold standard procedures for the treatment of inguinal hernia in adults. The extraperitoneal space of the lower abdomen and pelvis has a loose structure that can provide sufficient space for performing large area mesh reinforcement, making it an ideal approach in the treatment of inguinal hernias. TEP repair provides a minimally invasive, standardized, and refined repair with low hernia recurrence rates and morbidity. However, this type of endoscopic TEP surgery is traditionally limited to the treatment of inguinal hernias. We hypothesize that the concept of totally endoscopic anatomical repair may also be applied to treat other abdominal wall hernias.

We recently reported our experience performing totally endoscopic sublay (TES) repair for the treatment of midline ventral hernias [2]. Whereas TES requires long bilateral incision of the posterior rectus sheath, we learned that it might be possible to use the anatomical space between the peritoneum and the posterior rectus sheath (preperitoneal space) for reduction of the hernia sac, closure of the defect, and implantation of a large mesh. Based on this experience, we started a prospective study to develop a reliable technique for complete endoscopic totally extraperitoneal (preperitoneal) approach (TEA) in the treatment of primary midline ventral hernias. The presented study reports our preliminary results in 28 patients in who this novel technique was used from September 2017 to December 2019.

## Materials and methods

This prospective study was conducted in the Affiliated Hexian Memorial Hospital of Southern Medical University between September 2017 and December 2019 and was approved by the institutional ethical review board. The inclusion criteria were as follows: primary midline ventral hernias, including umbilical and epigastric hernias with or without concomitant mild rectus diastasis (< 3 cm). All patients had good preoperative general condition without significant comorbidities and were able to tolerate pneumoperitoneum and general anesthesia. Considering that TEA is a new technique with specific technical demands and not yet precisely defined learning curve, we set the following exclusion criteria: maximum hernia defect width > 4 cm, concomitant rectus diastasis with a width > 3 cm, BMI < 30, pregnancy, and patients with absolute clinical contraindications (e.g., active intra-abdominal infection or abdominal wall fistula). Preoperatively, all patients underwent whole-abdomen computed tomography (CT) to measure the hernia defect and aid with the operative planning. Complete medical history and physical examinations were obtained to identify any abnormalities that could affect surgery. All patients provided informed consent preoperatively.

Patient demographics, hernia size, intraoperative findings, operative details, and perioperative and postoperative data were collected prospectively. The main outcome of the study included operative difficulties, postoperative complication rates and hernia recurrences. Complications including hematoma, infection, seroma, wound dehiscence or any serious adverse event as well as postoperative pain scores were recorded and analyzed.

All patients underwent clinical examination 2 weeks after surgery and were subsequently followed up by a telephone interview every 2 months during the follow-up period. A routine postoperative CT scan was performed at the second-week clinic visit to detect any complications. During the follow-up period, if a problem was identified during the telephone interview, the patient was requested to visit the hospital for further examination.

In our study, continuous variables are presented as means/standard deviations, while categorical variables are presented as numbers/percentages.

### Operative technique

**Fig. 1:**
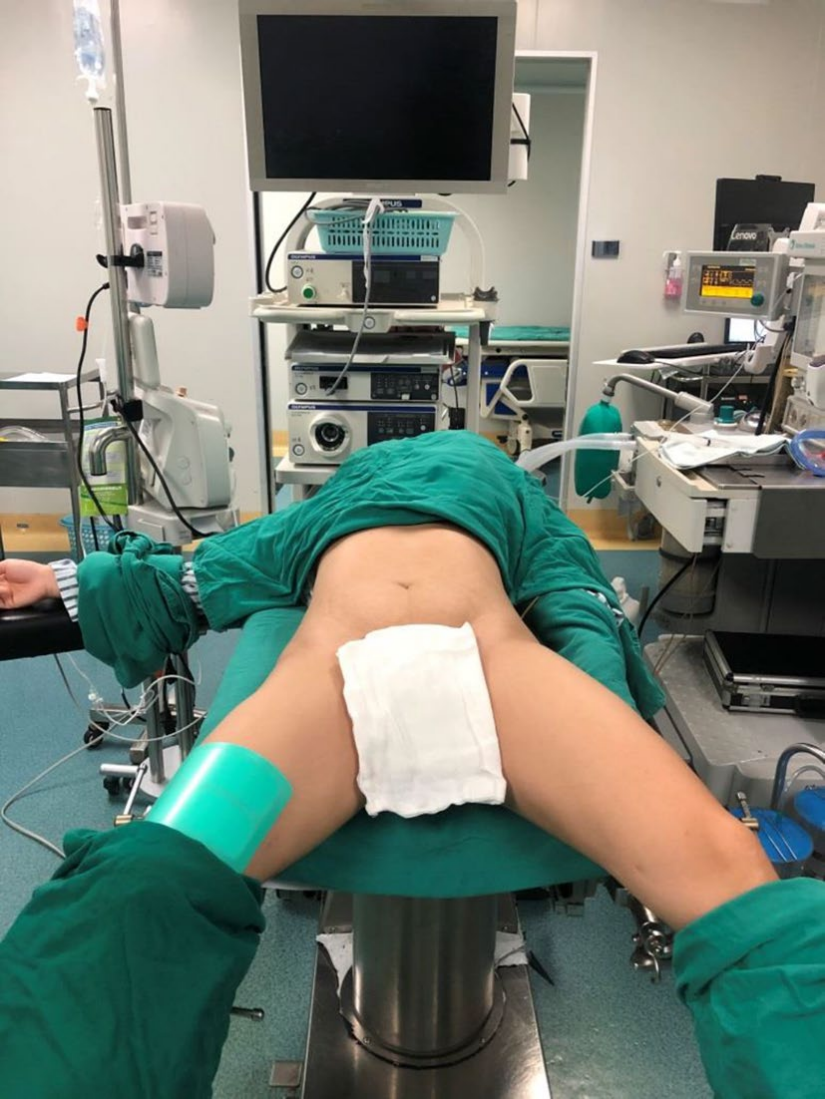
Position of the patient on the operating table

**Fig. 2:**
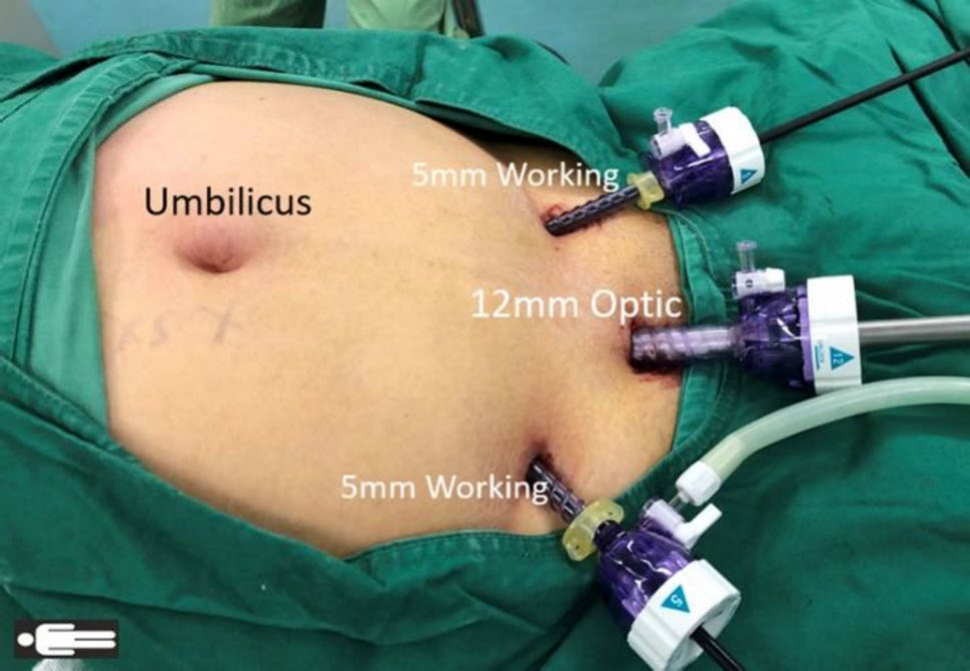
Primary extraperitoneal space establishment and port placement
Step 2: The extraperitoneal space around the arcuate line is dissected. During the cephalad dissection of the extraperitoneal space, care must be taken in the identification of the arcuate line and bilateral posterior sheaths (Fig. 3). The correct way to enter the true extraperitoneal space above the arcuate line is to create a dissection right below the posterior sheath. If the rectus muscle fibers are still visible during the dissection, it indicates that the correct plane of the retromuscular space has been violated. During the separation process, one hand is generally used to provide persistent and gentle downward retraction of the peritoneum, while the other hand prepares an electric hook to enable directed cautery or blunt dissection. Both hands can alternately hold the electric hook depending on which side is dissected. A small gauze can be introduced that is held by noninvasive forceps to compress the peritoneum. This helps to reduce the pressure on the peritoneum and decreases the risk of peritoneal tears during retraction.

After the initiation of general anesthesia, patients assumed a supine position with their legs apart, and a monitor screen was placed at the cephalad side (Fig. 1). Considering the prolonged operative time, a Foley catheter was routinely inserted preoperatively to decompress the Retzius space. Only routine laparoscopic instruments were required for the operation, including a 10-mm 30° laparoscope and ordinary 5-mm caliber instruments including a monopolar electrode hook used for dissection. No advanced energy instruments were required. The technical details of the operation are described step-by-step as follows:

Step 1: A preperitoneal space is established in the lower abdomen. The hypogastric preperitoneal space is established directly above the pubic area. A 12-mm transverse incision is created approximately 3 cm above the pubic symphysis. The subcutaneous tissue is incised and pulled apart using a pair of small hooks to expose the underlying anterior rectus sheath. The anterior sheath is incised approximately 10 mm transversely and lifted. Forceps are used to separate the underlying rectus muscle fiber; a small longer hook is subsequently pulled up through the opening of the anterior sheath, and a 12-mm disposable trocar is inserted into the preperitoneal space (below the arcuate line). Next, the preperitoneal space is initially established by endoscopic dissection, and bilateral separation reaches the attachments of the arcuate line of Douglas to the linea semilunaris. Care is taken to avoid any injury to the inferior epigastric vessels. The 5-mm working ports are inserted bilaterally. After the preliminary space and ports are established, the surgeon moves between the patient’s legs and prepares for subsequent cephalad dissection and extraperitoneal separation (Fig. 2).

**Fig. 3:**
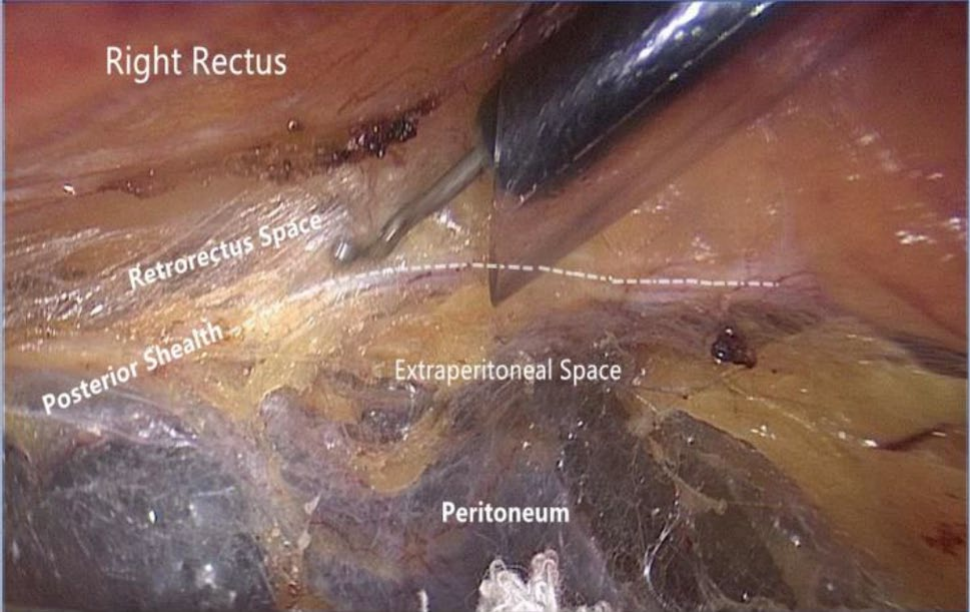
Identifying the posterior sheath and locating the correct extraperitoneal space
Step 3: The umbilicus with the surrounding preperitoneal area are dissected. The peritoneum in the area slightly above the arcuate line, which is posterior to the corresponding rectus muscles bilaterally, is tightly attached to the posterior sheath on the respective sides and is extremely thin. Gentle separation is required to prevent the violation of the peritoneum; otherwise, it will affect subsequent manipulations. For midline defects, the linea semilunaris on each side is the lateral boundary of the peritoneal dissection. If there is a Spigelian hernia or lateral defect such as a lumbar hernia, further lateral dissection can be performed. During dissection around the umbilicus, the atretic umbilical artery and urachus, which present as fibrous cord-like structures, will be encountered in the lower aspect of the umbilicus (Fig. 4). These structures can be directly severed using hook electrocautery. In some cases, there are recanalized blood vessels within these fibrous structures with diameters exceeding 2 mm; hemostasis of these vessels must be achieved with electrocoagulation before separation. In most circumstances, if an umbilical hernia contains the greater omentum or extraperitoneal fat, it can be reduced simultaneously during dissection (Fig. 5). The skin of the umbilicus must be carefully preserved, and constant palpation of the umbilicus during the dissection process can provide perception of the dissecting depth to prevent electrocautery-induced thermal injury of the umbilical skin. If the hernial sac is opened, it can be closed using an absorbable suture in a running fashion once the entire peritoneal dissection is accomplished.

**Fig. 4:**
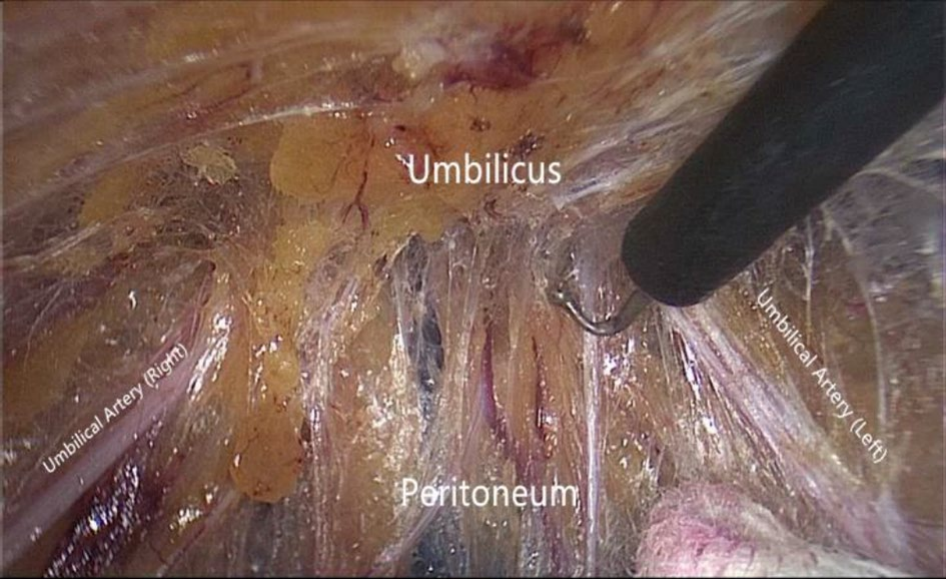
Cord-like structures visualized in the extraperitoneal space of the lower umbilical margin

**Fig. 5:**
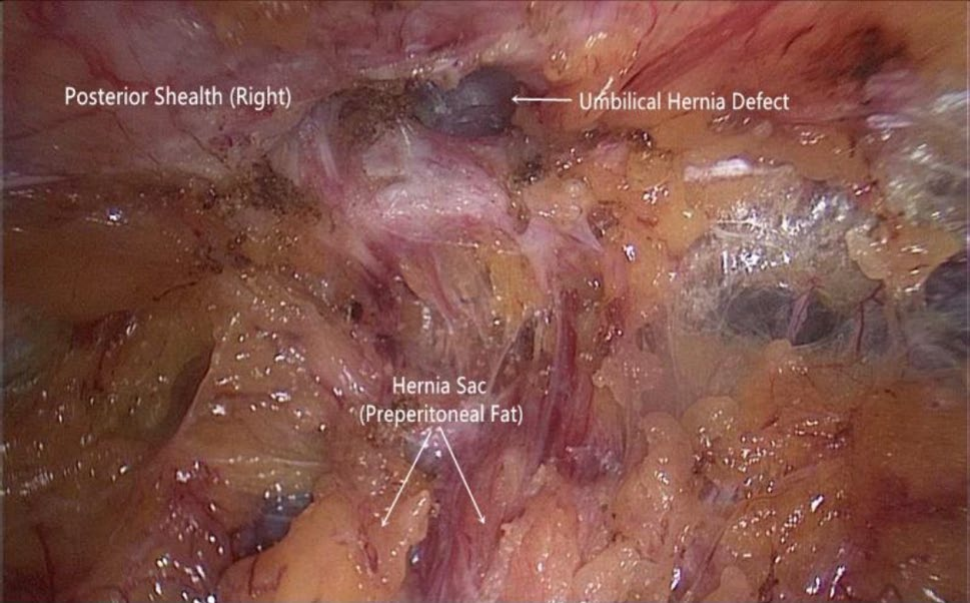
An umbilical defect was revealed after hernia content reduction
Step 4: The supraumbilical region is dissected. There are several fibrous cord-like structures above the umbilical ring, which are the inferior attachments of the falciform ligament to the umbilicus. If there is no varicose vein within these structures, they can be divided directly using electrocautery. In the supraumbilical region, extraperitoneal fat accumulates in the midline area, which makes it an ideal plane for separation. Conversely, in the lateral region, if there is limited fatty connective tissue between the peritoneum and its corresponding posterior sheath, both layers are attached more tightly. Thus, the midline area is generally dissected first and subsequently extended to create space toward its lateral margin. In case of an epigastric hernia, the hernial sac should be dissected as completely as possible and the contents gently reduced. Less extraperitoneal fat is located in the lower part of the linea alba, but as it is extended upward to the xiphoid area, the amount of extraperitoneal fat gradually increases. Specifically at the anchoring region of the falciform ligament, it accumulates and forms a fatty triangle pad; this is an important extraperitoneal anatomical safety landmark (Fig. 6). The superior boundary for dissection is located 5 cm above the topmost margin of the defect, whereas the lateral sides are bound by the linea semilunaris. If upward separation through the original two operation ports is out of reach, one or two additional 5-mm ports can be introduced on either side within the linea semilunaris.

**Fig. 6:**
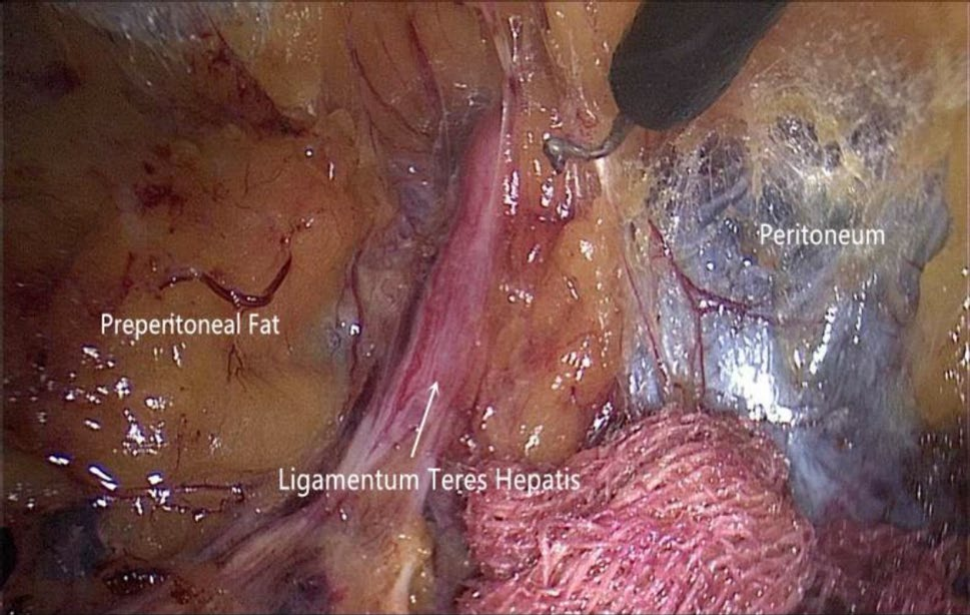
Extraperitoneal space in the subxiphoid area. The ligamentum teres hepatis is visualized
Step 5: The abdominal wall defect is closed, and any peritoneal fenestration is repaired. Once the peritoneal dissection is accomplished, the anterior abdominal wall defect is closed to eliminate the dead space and prevent postoperative seroma formation. This may be performed with transmural interrupted suturing using a suture passer or continuous intracorporeal suturing with a barbed 2–0 suture. We did not plicate the linea alba with coexisting rectus diastasis. Peritoneal defects created by previous dissection should be closed and are generally sutured using a 2–0 absorbable suture in a running fashion. Of note, suturing of the peritoneum must be performed carefully; otherwise, the fenestration will widen due to tearing.
Step 6: The mesh is placed. The mesh should extend beyond the defect at least by 5 cm in all directions. In our series the maximal defect size of repaired umbilical hernias was 4 cm or less, which could adequately be repaired with a commercially available 15 × 9- cm^2^ self-gripping mesh (ProGrip™; Covidien, France). The mesh is rolled up and inserted through the 12-mm port. Once in place, the mesh is unrolled with the grip side facing upward, lifted, and attached to the posterior sheath (Fig. 7). However, in patients presenting with an epigastric hernia, especially when combined with a rectus diastasis demonstrating a weak fascial structure, it seemed to be advisable to enforce the weakened anterior abdominal wall in the midline. Therefore, in these cases a larger mesh (20 × 10-cm^2^)—a long rectangular macroporous polyvinylidene fluoride mesh (DynaMesh®-CiCat; FEG Textiltechnik mbH, Germany)—was selected not only to cover the epigastric defect but also the weakened linea alba inclusive the umbilical area. In this setting, a chemical adhesive glue (Compont®; COMPONT Co. Ltd., China) was used for mesh fixation. Traumatic fixation such as tackers or transfascial stitches is unnecessary.

**Fig. 7:**
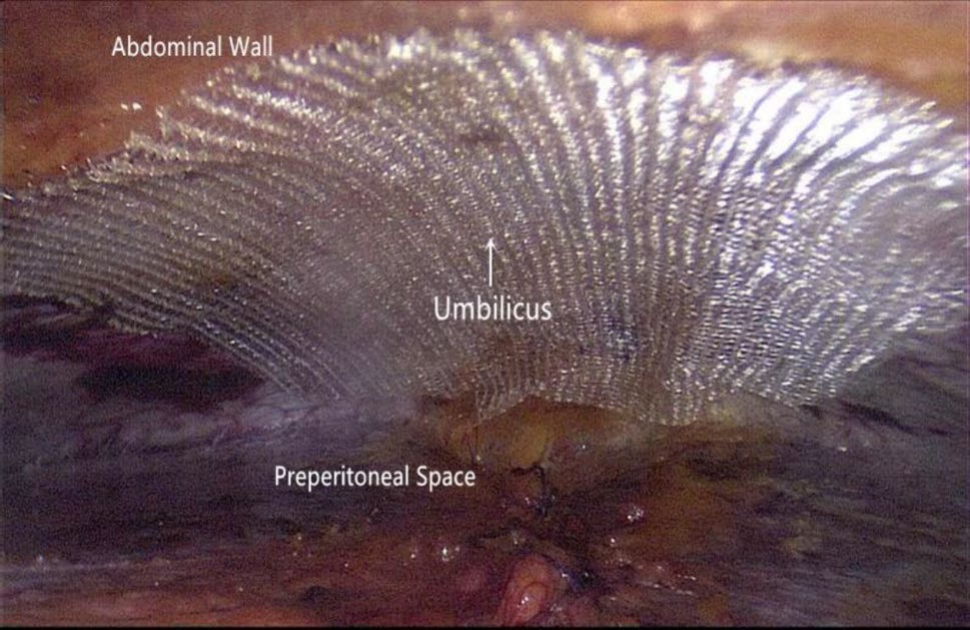
Placement of a 15 × 9-cm^2^ self-gripping mesh in the extraperitoneal space (a case of umbilical hernia)
Step 7: After mesh placement, the entire surgical field is carefully re-examined. Drainage is not imperative if the field is hemostatic and clean. The retropneumoperitoneum is removed, the wounds are closed with subcutaneous absorbable sutures, and an abdominal binder is placed.

## Results

Twenty-eight patients (10 men and 18 women; age 33–70 years) participated in this study. All operations were successfully performed without serious intraoperative complications or conversion to open surgery. The mean operative time (skin to skin) was 103.3 ± 14.1 min (range 85–145 min). The postoperative diagnoses were umbilical hernia in 17 patients, primary epigastric hernia in 6, umbilical hernia concomitant with epigastric hernia in 3, umbilical hernia concomitant with mild rectus diastasis in 1, and epigastric hernia concomitant with mild rectus diastasis in 1. Patient’s general demographics and perioperative data are listed in Table 1. All the anterior defects were closed using a 2-0 barbed suture. For the 17 patients with primary umbilical hernia, a 15 × 9-cm^2^ self-gripping mesh was used to repair the defect, while for the rest of the patients, we used long rectangular 20 × 10-cm^2^ macroporous polyvinylidene fluoride mesh to reinforce the weakened linea alba. Intraoperative bleeding was minimal. Postoperative pain was qualitatively mild with the average pain visual analogue scale score under physical stress of 1.9 ± 0.6 (range 1–3) on the first postoperative day. None of the patients required intravenous analgesics. Patients were allowed an oral diet and early ambulation after recovering fully from the anesthesia. The average postoperative length of hospital stay was 1.9 ± 0.6 days (range 1–3 days).

**Table 1:** Patient’s general demographics and perioperative data

Postoperative diagnoses	UH	EH	UH and EH	EH with RD/UH with RD	In SUM
*N*	17	6	3	1/1	28
Age (years)	50.4 ± 10.0	48.3 ± 11.8	49.3 ± 5.7	66/45	50.2 ± 9.9
BMI (kg/m^2^)	26.6 + 1.9	26.7 ± 4.6	24.7 ± 0.4	24.6/21.5	26.2 ± 2.7
ASA score	1.4 ± 0.5	1.5 ± 0.8	1.0 ± 0.0	2/1	1.4 ± 0.6
Sex (male/female)	6/11	1/5	2/1	Male/female	10 (35.7%)/18 (64.3%)
Operative time (mins)	100.6 ± 14.8	107.2 ± 10.0	117.7 ± 11.7	95/90	103.3 ± 14.1
Estimated blood loss (mL)	13.2 ± 5.6	11.7 ± 2.6	15.0 ± 0.0	10/10	12.9 ± 4.6
Defect width (cm)	2.4 ± 0.8	2.3 ± 0.4	1.8 ± 0.3, 2.2 ± 0.3	1.5/3	2.3 ± 0.6
Defect area (cm^2^)	6.1 ± 4.3	5.2 ± 2.1	3.4 ± 1.0, 4.8 ± 1.3	2.25/9	4.9 ± 2.5
Mesh type	ProGrip	CiCat	CiCat	CiCat	
Mesh area (cm^2^)	135	200	200	200/200	
Mesh fixation with glue	–	Glue	Glue	Glue	
LOS (days)	2.0 ± 0.7	1.7 ± 0.5	2.0 ± 0.0	2/2	1.9 ± 0.6
(VAS)	1.9 ± 0.8	1.8 ± 0.4	2.0 ± 0.0	2/2	1.9 ± 0.6
Follow-up (months)	19.1 ± 5.5	15.5 ± 5.5	21.3 ± 3.5	11/12	18.0 ± 5.6

Because the RD patients are limited in number, we list the data individually

*LOS* length of postoperative stay, *VAS* pain score under physical stress on 1st postoperative day, *UH* umbilical hernia, *EH* epigastric hernia, *RD* rectus diastasis

By December 2019, 28 patients had been followed up for a mean period of 18.0 ± 5.6 months. Readmissions within 30 days or recurrences were not observed in this series. All patients recovered uneventfully without serious postoperative complications. Two patients had mild localized seroma formation that persisted for approximately 4 weeks and subsequently resolved spontaneously. One patient experienced an episode of suprapubic 12-mm wound dehiscence; after 1 week of daily wound care, it was sutured to achieve delayed primary closure and healed successfully.

## Discussion

Multiple approaches and techniques utilizing different layers of the abdominal wall and surgical methods can be used in hernia repair, of which retromuscular repair (sublay) and intraperitoneal onlay mesh (IPOM) repair are most commonly used [3]. Although laparoscopic IPOM surgery is technically simple and minimally invasive [4], prosthetic material placement in the abdominal cavity carries risk of adhesions and visceral complications and requires invasive fixation, which poses a series of postoperative risks [5–7]. In theory, the sublay position utilizing the posterior space of the rectus abdominis is a more ideal layer for mesh augmentation [8]. However, conventional open sublay surgery requires a large midline incision, which may involve complications such as large tissue trauma, postoperative pain, and wound infection. Several novel extraperitoneal techniques have been developed to perform retromuscular repair through minimally invasive methods, including mini/less open sublay (MILOS) repair [9], followed by modified MILOS surgery with endoscopic assistance (e-MILOS) [10], laparoscopic transabdominal retrorectus repair [11], endoscopic transabdominal midline reconstruction technique facilitated by endoscopic linear stapling [12], completely endoscopic sublay dissection methods including the enhanced-view totally extraperitoneal (eTEP) technique initiated by Belyansky et al. [13], and the TES repair technique reported by our team [2].

To connect the bilateral retrorectus spaces across the midline with subsequently placement of a large mesh in the sublay plane to reinforce the impaired anterior abdominal wall, the medial border of the bilateral posterior sheaths must be incised longitudinally and subsequently reconnected by a running suture or stapler technique. This increases the surgical complexity and prolongs the operative time. Certainly, as early as 2002, Miserez et al. [14] described the first endoscopic retromuscular dissection technique in which the retromuscular plane on both sides was developed and connected. The crossing of the midline is accomplished by incising the rectus sheath on both sides and dissecting down the peritoneum behind the linea alba. However, Miserez’s technique was not widely adopted, likely due to its technically challenging and complex nature. Moreover, whether the violation of posterior sheaths will affect the future stability and strength of the linea alba remains unknown.

Furthermore, there is a justifiable concern whether this kind of abdominal wall reconstruction is appropriate for small- to moderate-sized primary midline defects such as umbilical and upper epigastric hernias. Certainly, in our practice of performing totally endoscopic sublay repair TES (2) when starting the dissection in the suprapubic retroperitoneal space (Retzius), we found that after identification of the arcuate line it seemed to be possible to enter the anatomical layer between the peritoneum and posterior rectus sheath without violation of the fascial structures. Although this preperitoneal space may be rather delicate above the arcuate line and may contain insufficient connecting tissue, it can usually still be carefully and meticulously separated, establishing a sufficient extraperitoneal (preperitoneal) pocket to accommodate a large non-coated mesh for hernia repair. Several surgeons have tried to use this extraperitoneal plane for optimal mesh placement and such to avoid any violation of the rectus sheath or the linea alba [15–17]. However, in all of these studies, the transabdominal route for approaching this extraperitoneal space was used. It may be easy to dissect this space [18], if it contains a substantial amount of connective and fatty tissue, but in quite a few patients, the peritoneum is significantly thin, and there are tight adhesions to the posterior rectus sheath. Therefore, several surgeons intended to dissect this plane for placement of the mesh, but in most of their cases, the rectus sheath was opened and the mesh was placed in the retrorectus position [19, 20]. Recently, it was shown that a robot-assisted dissection technique may be beneficial [21, 22], but in this technique, a transabdominal approach with all its risks is required so far. However, to the best of our knowledge, the presented study shows for the first time that in selected patients a complete endoscopic repair with placement of a large mesh into the extraperitoneal (preperitoneal) space is feasible, safe, and cost-effective and provides a reliable and standardized repair.

A limitation of the described totally extraperitoneal approach (TEA), may be that the surgical technique may be demanding in dependence on the individual structure of the abdominal wall layers. Meticulous dissection along the posterior sheath of the rectus muscle is essential; otherwise, the sheath may be injured, and conversion to a sublay operation becomes necessary. Especially important, in the lateral upper abdominal wall the peritoneum is relatively thin, and in the umbilical region there might be tight adhesions to the linea alba, in so far dissection is difficult and the posterior rectus sheath may be easily fenestrated. Dissection of these areas requires gentle and meticulous technique. In so far patients presenting with an incisional hernia or after open lower abdominal surgery are not suitable for TEA and excluded for performing this technique. In order to diminish the risk for peritoneal tears it may be helpful to compress the peritoneum downwards using some pad to provide a stable, moderate amount of tension which is favorable for dissection. Regarding the dissection instrument, we believe that the monopolar electrode hook is an ideal device for accurate surgical dissection. Finally, the extent of separation must exceed the defect by at least 5 cm. In our study, the association between the mesh area and defect size area was according to the guidelines for the prevention of a recurrence, which was far more than 16:1 [23, 24].

In some difficult cases, accidental tearing of the peritoneum during dissection seems inevitable. In this scenario, the operator should shift the dissecting plane from the extraperitoneal to the retromuscular space. In some cases if required, a small segment of the posterior sheath being cut down and remaining at the peritoneum is acceptable. Once the operator passes the area at risk for peritoneal lesion, the dissection plane may shift back to the extraperitoneal plane. Anyway, at the end of the dissection, any openings of the peritoneum should be closed by suture.

Our preliminary experience suggests that the optimal indication for TEA surgery is small- to moderate-sized primary umbilical and epigastric hernias (defect width up to 4 cm) in non-obese patients. When developing this new technique, to avoid some harm for the patient, we followed strong inclusion criterions. However, with increasing experience most of the primary ventral hernias may be suitable to be corrected based on the TEA concept, including Spighelian and lumbar hernia. Undoubtedly, in an epigastric hernia that is extremely close to the xiphoid process, a long distance extraperitoneal dissection from the suprapubic area to the xiphoid area will require a lot of dissection and seems to be unreasonable. In these cases, some surgeons recommend the transabdominal approach to enter the preperitoneal space [6]; however, an e-MILOS or eTEP technique seems to be more appropriate in this scenario than any other techniques [10, 13]. For a congenital lumbar hernia that is herniated from the superior lumbar triangle (Grynfeltt hernia), a retroperitoneal endoscopic approach can be utilized to achieve extraperitoneal repair [25]. Theoretically, if dissected in an appropriate anatomical plane, the peritoneal structure derived from the embryonic endoderm can be entirely separated from the parietal structure that is derived from the mesoderm; therefore, acting through the totally visceral sac separation (TVS) concept, extraperitoneal repair with a non-coated mesh can be achieved for these kinds of primary ventral hernias.

According to our experiences TEA has the following strengths. (1) It is a totally endoscopic operation that complies with the principles of minimally invasive surgery. (2) It is a reliable and reproducible method that does not require a specific device, making it cost-effective. (3) It uses a natural surgical plane without violating the normal anatomical structures, thereby conforming to the intrinsic anatomical principles and dramatically reducing postoperative pain. (4) The mesh is excluded from the intraperitoneal cavity, eliminating the risk of intraperitoneal foreign body-related complications. (5) An expensive anti-adhesion mesh or traumatic fixation tacker is not necessary, thereby significantly reducing the healthcare cost.

A further limitations of the TEA, beside that this technique is considerably challenging, may be that its current indication is limited to small and moderate defects in primary ventral hernias of the abdominal wall.

In conclusion, our results suggest that in selected patients TEA is a safe and feasible minimally invasive alternative for the treatment of primary ventral hernias. It complies with the anatomical structure and functional demands of the abdominal wall, which may reduce the surgical trauma and possible postoperative complications. Expensive anti-adhesion mesh and fixation tacker are not required, which makes TEA more cost-effective. However, TEA is still in its early stages of development, and further studies are required to verify its role in the repair of ventral hernias.
